# Canine infantile left ventricular noncompaction

**DOI:** 10.1186/s12917-020-02480-7

**Published:** 2020-07-23

**Authors:** Maria Vilcu, Iuliu Scurtu, Dan G. Ohad, Ionel Papuc, Laura Scurtu, Flaviu Tabaran

**Affiliations:** 1 University of Agricultural Science and Veterinary Medicine, Calea Manastur 3-5, 400372 Cluj- Napoca, Romania; 2 The Koret School of Veterinary Medicine, Robert H. Smith Faculty of Agriculture, Food and Environment, Hebrew University of Jerusalem, P.O. Box 12, 76100 Rehovot, Israel; 3 Modis Competence Center, Strada Muresului 9, 400598 Cluj-Napoca, Romania

**Keywords:** Left ventricle noncompaction, cardiomyopathy, tricuspid valve dysplasia, pulmonary valve stenosis, dog

## Abstract

**Background:**

Left ventricular noncompaction (LVNC) is a rare form of cardiomyopathy currently described in humans and cats. It consists of a spongy myocardium characterized by prominent trabeculation and deep recesses involving more than 50% of the ventricular thickness. We describe the clinical and pathological features of LVNC combined with tricuspid valve dysplasia, double-orifice tricuspid valve and severe pulmonary stenosis in a puppy. In addition, we briefly review the LVNC causes, pathogenesis, forms and current diagnostic criteria.

**Case presentation:**

A seven-week-old intact German Shorthaired Pointer-cross male was presented with a poor body condition, exercise intolerance and dyspnea. Clinical exam identified a bilateral systolic murmur (grade IV/VI over the right heart base and grade III/VI over the left heart base). Echocardiography identified tricuspid valve dysplasia, mild mitral regurgitation, and severe pulmonic stenosis with a trans-valvar systolic pressure gradient of 106 mmHg. Left ventricular noncompaction was diagnosed by necropsy and further confirmed histopathologically by the presence of two distinct myocardial layers: an inner noncompacted zone covering more than 50% of ventricular thickness containing prominent trabeculation and deep recesses, and an outer zone of compact myocardium.

**Conclusions:**

This is the first case describing LVNC in a canine patient, supporting the introduction of this form of heart disease as a differential diagnosis for cardiomyopathies in juvenile and adult dogs.

## Background

Left ventricular noncompaction (LVNC), also known as “spongy myocardium” [1] or left ventricular hypertrabeculation [2, 3], a distinct form of cardiomyopathy with a highly variable but potentially aggressive course, was first described in humans in 1969 [2, 4]. It is morphologically characterized by deep recesses and prominent myocardial trabeculation involving more than 50% of the myocardial thickness. It was recently described as a spontaneous cardiac pathology in cats [5, 6].

In a normally developed mammalian myocardium, two concentric myocardial layers are present: a compacted, thick outer layer and a noncompacted inner layer [4, 6]. A full-thickness spongy trabeculated ventricular myocardium is normally found in adult fish, amphibians and reptiles, as well as until the late mid-term embryonic stage of birds and mammals [2].

In mammals, embryonic heart development consists of four distinct stages. In the initial stage, the heart is composed of a single-cell layer of myocardium and a single-cell layer of endocardium. The second stage of development starts at early mid-gestation, when a two-layered myocardium (an inner area of trabeculated myocardium and a compact outer layer) is formed [7]. The formation of myocardial trabeculation occurs as an attempt to increase the endocardial surface-area and to enhance gas exchange before the maturation process of the coronary circulation is completed [6]. The third stage of cardiac development consists of myocardial compaction, during late mid-gestation [7]. This stage is associated with trabecular remodeling and compaction of the left ventricle (LV) and occurs simultaneously with myocardial “invasion” of the developing coronary circulation [4]. During this stage, the transformation of intertrabecular recesses into capillaries leaves a relatively smooth ventricular endocardial surface [1, 6]. Trabecular compaction is normally more advanced in the left ventricular than in the right ventricular myocardium [6]. In the case of noncompacted cardiomyopathy, the failure of the in-utero myocardial compaction and trabecular fusion process is responsible for the typical persistence of a post-natal spongy or hypertrabecular myocardium. The fourth stage occurs during the late fetal and neonatal phase and represents the formation of a mature and multilayered spiral myocardium [7].

The pathogenesis of LVNC is thought to be associated with a failure of the in utero myocardial compaction process. This hypothesis is supported by new evidence of an autosomal dominant form of LVNC in humans, caused by a mutation in the mindbomb homolog 1 (MIB1) gene encoding a protein, which regulates cell proliferation and compaction of the fetal myocardium. Several other hypotheses attempt to explain the persistence of LV trabeculation into adulthood, such as an adaptation to special hemodynamic conditions, an inadequate adhesion of cardiac myocytes, cardiac neuropathy, or a disturbed cardiac conduction system comprising His and Purkinje’s fibers [3]. Mutations associated with LVNC have been reported in more than 40 human genes, including genes encoding for sarcomeric (MYBPC3, MYH7), ion channel (SCN5A, HCN4), cytoskeletal (LDB3/Cypher/ZASP, LMNA), and chaperon proteins [2, 8, 9].

We describe the clinical and pathological findings of an unusual form of congenital heart disease in a dog, consisting of LVNC concomitant with tricuspid valve dysplasia (TVD), double-orifice tricuspid valve and pulmonic stenosis (PS).

## Case presentation

A seven-week-old intact male German Shorthaired Pointer-cross, weighing 3.3 kg with a history of poor body condition, anorexia, reluctance to move and dyspnea, was presented to the Cardiology Clinic of the University of Agricultural Science and Veterinary Medicine, Cluj-Napoca, for further evaluation.

On presentation, the dog was moderately dyspneic and lethargic, with a body condition score of 3/9 and tachycardia (230 bpm). A grade IV/VI right basilar systolic and a grade III/VI left basilar systolic heart murmur was present.

Electrocardiographic findings included a sinus tachycardia with a heart rate of 230 bpm, P pulmonale (0.5 mV, Lead-II) and a normal mean electrical axis of the ventricular depolarization process.

Standard transthoracic two-dimensional and Doppler echocardiography was performed in right and left lateral recumbencies using a 10 MHz, phased array transducer, according to ACVIM recommendations [10]. In the right-parasternal, long-axis, four-chamber view, a congenital TVD was identified with severe right atrial enlargement. The tricuspid valve annulus was displaced ventro-caudal, towards the right ventricular (RV) apex. A voluminously appearing tricuspid insufficiency jet with a 4.69 m/s peak flow velocity, along with mild mitral regurgitation was present. A severe, type B pulmonic stenosis [11] with valvular dysplasia, severe thickening of the valve cusps and a hypoplastic valve annulus was also demonstrated. The peak flow velocity across the stenosis was 5.16 m/s. There was moderate to severe post-stenotic pulmonary arterial dilatation. There was generalized concentric RV hypertrophy, presumably, secondary to the severe PS. Aortic flow was laminar and its velocity was normal at 1.29 m/s.

Due to the severe cardiac pathology, the dog was humanely euthanized with the owner’s consent and necropsy was allowed. For euthanasia we used T-61®, which was intravenous administered (0.5 ml/kg). Before euthanasia, the dog was anesthetized with a combination of xylazine (1 mg/kg, i.v.) and ketamine (10 mg/kg, i.v.).

**Fig. 1:**
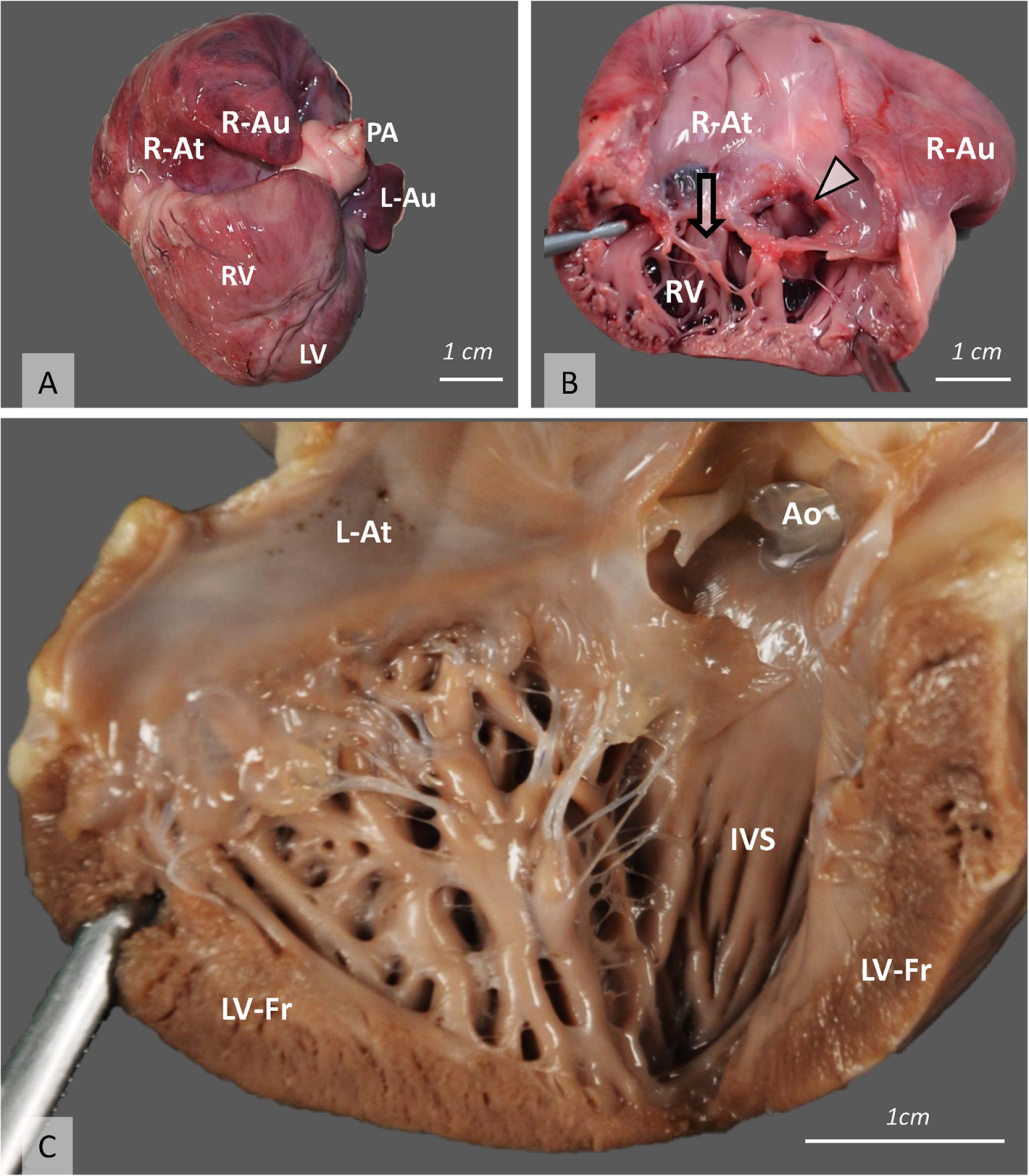
Gross pathology of the heart. **a** Severe dilation of the right atrium, right atrial appendage and right ventricle, as seen from the right. **b** An inside view of the right heart showing severe dilation of the right atrium with a dysplastic tricuspid valve consisting of marked hypoplasia of the chordae tendineae, and thick, deformed, and malpositioned valve leaflets. Note the large accessory orifice (arrowhead) of the double-orifice tricuspid valve. The arrow indicates the normal right ventricular inflow tract. **c** An inside view of the left heart demonstrating the prominent trabeculation of the left ventricular free wall and a marked hypoplastic and branched papillary muscle (Ao, aorta; IVS, interventricular septum; L-At, left atrium; L-Au, left atrial appendage; LV, left ventricle; LV-Fr, left ventricular free wall; PA, pulmonary artery; R-At, right atrium; R-Au, right atrial appendage; RV, right ventricle)

Necropsy revealed moderate hepatomegaly with diffuse hepatic steatosis. Within the gastrointestinal tract, multifocal ulcerative gastritis was present. Gross examination of the heart revealed marked right-atrial dilatation (Fig. 1a) with TVD, consisting of marked hypoplasia of the chordae tendineae, tricuspid valve leaflet thickening, deformation and malposition. A large, ovoid, accessory orifice with smooth margins (measuring 0.9 cm) of the tricuspid valve was present. The accessory orifice corresponded to the third type of double-orifice tricuspid valve (DOTV), which is an extremely rare form of tricuspid anomaly in humans [12–14]. The DOTV involved the septal leaflet and it had its own subvalvular apparatus (Fig. 1b).

Pulmonary valve stenosis and dysplasia with post-stenotic dilatation of the pulmonary trunk were confirmed. Within the ventricles, multiple recesses and elaborate trabeculation were projecting through both the apical and middle ventricular areas, with the LV being more severely affected. The non-compacted layer was mainly on the free walls, but also partially extending into the interventricular septum, in both the middle and the apical regions (Fig. 1c).

The entire heart was fixed in 10% neutral buffered formalin, trimmed in a transversal plane, and multiple samples were routinely embedded in paraffin, sectioned, and finally stained with hematoxylin and eosin.

**Fig. 2:**
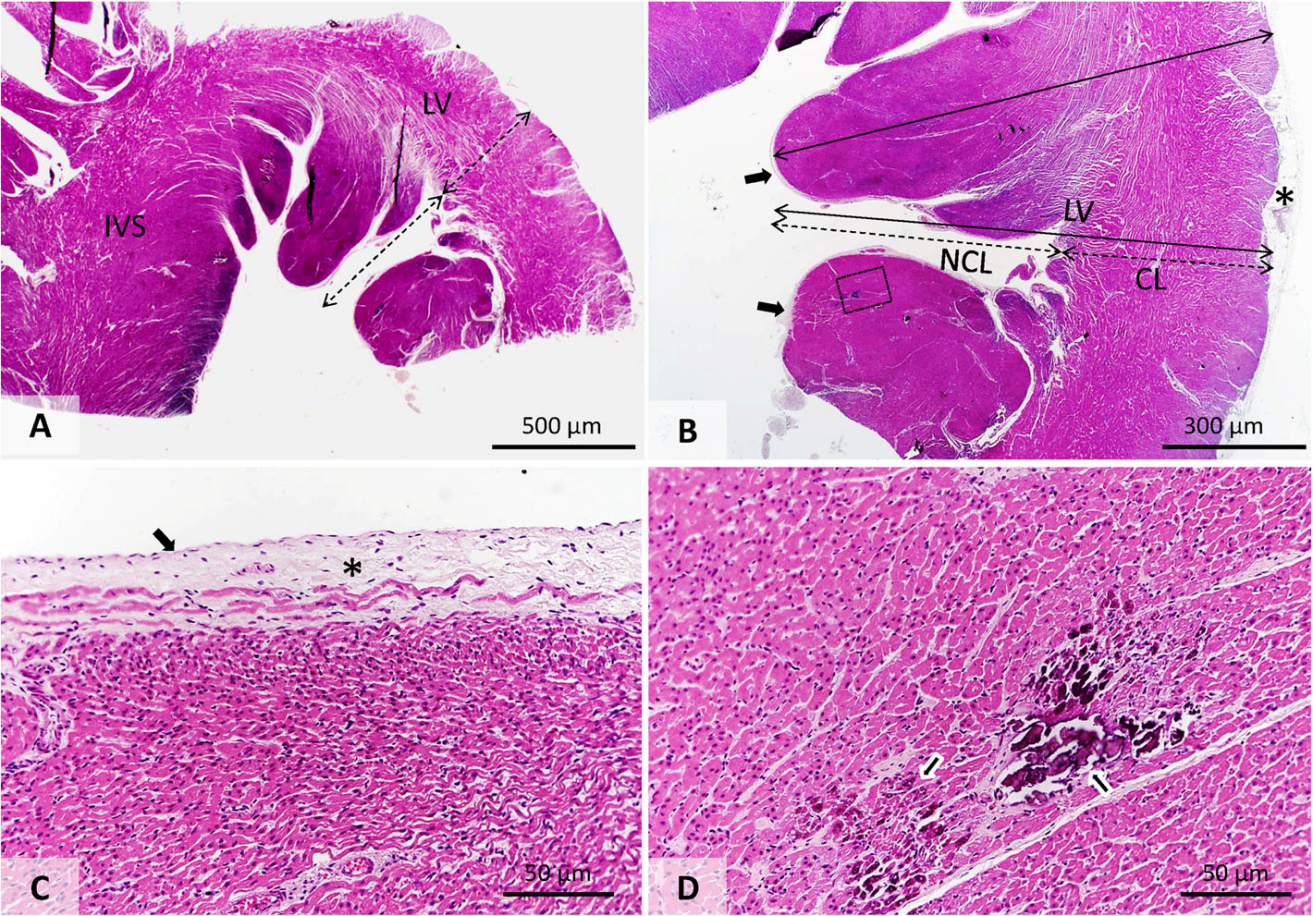
Histopathologic features of the left ventricular free wall and interventricular septum **(a, b)** Left ventricular free wall: multiple papillary trabeculae with a broad basis, an increased basophilia and a thick fibrous endocardium. Note that the noncompacted layer is thicker than the compacted one. **c** Prominent subendocardial fibrosis (asterisk). **d** Detailed image of the demarked area from image B. The arrows indicate several well-delineated areas of dystrophic mineralization (CL, compacted layer; IVS, interventricular septum; LV, left ventricular free wall; NCL, non-compacted layer)

Microscopically, the recesses appeared between multiple papillary trabeculae with a broad basis, communicating with the LV cavity. There were no elements identified to support the communication between the recesses and the coronary arteries. The entire LV cardiac wall thickness, including the noncompacted and compacted areas of both the free and the interventricular septal walls, was measured perpendicularly from the endocardial surface to the epicardium. The ratio between non-compacted and compacted areas was more than 50% (Fig. 2a and a). The same measurements were conducted on the RV myocardium, the ratio of which was even higher at 75%. Other histopathological findings were represented by diffuse subendocardial fibrosis and multifocal dystrophic mineralization of the left ventricle myocardium (Fig. 2c). These findings are consistent with the diagnosis of LVNC [3, 15] and include RV noncompaction involvement [15].

## Discussion and conclusions

While recognized as a relatively common form of cardiomyopathy in humans, ventricular noncompaction has only been reported as a spontaneously occurring pathology in cats. In two independent reports, LVNC was described in a Maine Coon-cross cat that was heterozygous for the cardiac myosin binding protein C mutation (A31P), in 2017 [6], and in a two-month-old kitten affected by Purkinje fiber dysplasia, in 2009 [5]. Experimentally, LVNC is being studied in transgenic mouse models and in species in which myocardial trabeculation is ordinarily present, such as chicks and adult fish [2, 7, 8, 16–20]. To the author’s best knowledge, this is the first report of LVNC in a canine patient.

Depending on the anatomical distribution, age of clinical onset, and coexistence of other congenital cardiac anomalies, several forms of myocardial noncompaction are described in humans. Based on the ventricle involved, myocardial noncompaction is classified as either left ventricular or biventricular (BVNC), where, in addition to LVNC, RV involvement is confirmed by a ratio of more than 75% between the noncompacted and compacted layers. Left ventricular noncompaction is classified as congenital or acquired (or as pediatric and adult form, respectively), or as isolated (or simple) versus non-isolated (i.e., associated with other cardiac anomalies) [2].

Based on genetics and the typical disease natural history, pediatric LVNC is likely a distinct, primary condition while the adult onset LVNC is a phenotypic variation of other cardiomyopathies [4]. In children, the LVNC is often associated with atrial septal defect, ventricular septal defect, patent ductus arteriosus, Ebstein’s anomaly, atrioventricular and pulmonary valvular abnormalities, ventricular septal defects, persistent left superior vena cava, histiocytoid cardiomyopathy, partial anomalous pulmonary venous return, and coronary ostial stenosis [15, 21].

The present LVNC case revealed several congenital cardiac co-morbidities, similar to what was reported in children. These co-morbidities involve both atrioventricular (tricuspid valve dysplasia) and semilunar valve defects (pulmonic stenosis).

While historically reported only based on gross pathology and histopathological findings, the diagnosis of LVNC is currently mainly attained by echocardiography [3, 22, 23] and, more recently, by cardiac MRI [1, 24–28], which is likely the reason behind the higher incidence of LVNC reported in recent years. Characteristic LVNC echocardiographic features include: (1) a thick, bilayer myocardium, (2) prominent trabeculation, and (3) deep endomyocardial recesses [23]. In the 8-week-old Savannah kitten with Purkinje fiber dysplasia/histiocytoid cardiomyopathy, LVNC was an incidental necropsy finding, with no pre mortem echocardiography performed. In the Maine Coon cross cat, the LVNC was seen on echocardiography at two years of age, as a “moth-eaten” appearance of the LV wall in short-axis view with deep recesses in the inner LV wall on the long axis view, representing hypertrabeculation. This was also subsequently confirmed histopathologically [6]. The cat had biventricular noncompacted cardiomyopathy where the RV was considered to be noncompacted, based on the LVNC criteria.

In the present case, the noncompacted layer of the RV comprised more than 75% of the wall, which corresponds to the biventricular form. However, definitively diagnosing biventricular noncompacted cardiomyopathy in this case was challenging for several reasons: (1) RV hypertrophy induced by the concurrent pulmonic stenosis, (2) trabeculation in the RV was an expected finding in the normal RV myocardium [4], and (3) due to the absence of a specific location compared with LVNC.

In this case, as in the one with Purkinje fiber dysplasia, the diagnosis was only based on gross and histopathological findings that met criteria such as a two-layer structured myocardium, segmental rather than scattered trabeculation within the apical LV, a NC/C ratio > 50%, and the concomitant presence of other congenital heart defects, namely TVD and PS. A disturbed cardiac conduction system comprising His and Purkinje’s fibers [3] can be potentially relevant to LVNC in the cat reported with Purkinje fiber dysplasia [5]. However, in the present canine case there was no evidence of Purkinje fiber involvement. The absence of echocardiographic evidence of LVNC in these two cases is due to the unknown existence of this pathology before 2009 in cats and present in dogs. In the Maine Coon cross cat, the presence of the cardiac myosin binding protein C mutation implied a more detailed examination of the myocardium. Therefore, the serendipity of identifying the noncompaction during echocardiography was superior in this case. In the present case, the size and the young age of the dog associated to the moderate dyspnea limited our ability to describe the specific features related to LVNC on the initial echocardiographic examination.

The three most common complications of LVNC are heart failure, ventricular arrhythmias and systemic embolic events, a so-called “classic triad” [29]. However, the clinical presentation of LVNC is highly variable [4]. Therefore, in the absence of this classic triad, the presence of only non-specific clinical signs such as poor body condition, anorexia, exercise intolerance and dyspnea, and the scarcity of cases reported in the literature with no widely accepted or robust diagnostic criteria, the diagnosis of LVNC is difficult to confirm.

In humans, the differential diagnosis list includes hypertrophic cardiomyopathy and dilated cardiomyopathy. In hypertrophic cardiomyopathy, the NC/C ratio does not usually reach a value higher than 2:1, and the trabeculated regions tend to be segmental in LVNC and diffusely distributed in LV hypertrophy [30]. In the present case, there was no pre or post mortal evidence of dilated cardiomyopathy, and the generalized concentric hypertrophy of the RV was attributable to the PS. While early manifestation of HCM could not be definitively ruled out in this patient, this disease entity is very rare in the dog and our echocardiographic findings were not characteristic for HCM.

Anomalies of the tricuspid valve in humans include tricuspid stenosis, Ebstein’s anomaly, tricuspid valve dysplasia, and double orifice tricuspid valve (DOTV). The latter is a rare congenital cardiac anomaly with three described variants: the “commissural” (the accessory ostium lying within the commissure); the central or “bridge type” (a fibrous bridge dividing the orifice into two ostia), and the “hole” (the accessory ostium lying within a leaflet) [12].The hole type should be distinguished from a simple fenestration or cleft which has no subvalvular apparatus. Usually DOTV is considered as benign and its pathophysiology is determined by concomitant lesions. Most cases are diagnosed incidentally during open-heart surgery or autopsy and it usually coexists with concomitant cardiac anomalies. The occurrence of DOTV is extremely rare and difficult to diagnose by echocardiography; therefore, the condition can be easily overlooked [12–14, 31, 32]. The identification of the accessory orifice in the septal leaflet of the tricuspid valve in the present case is compatible with the “hole” variant anomaly described in humans, reflecting the first confirmation of this pathology in a canine patient.

In conclusion, this is the first description of LVNC in a dog, supporting the introduction of ventricular myocardial noncompaction as a differential diagnosis for canine cardiomyopathies

## Abbreviations

DOTV: Double-orifice tricuspid valve
LV: Left ventricle
LVNC: Left ventricular non-compaction
MRI: Magnetic resonance imaging
NC/C: Non-compacted to compacted myocardium
PS: Pulmonic stenosis
RV: Right ventricle
TVD: Tricuspid valve dysplasia

## Availability of data and materials

The datasets used and/or analyzed during the current study are available from the corresponding author on reasonable request.
